# Combined Cognitive Training vs. Memory Strategy Training in Healthy Older Adults

**DOI:** 10.3389/fpsyg.2016.00834

**Published:** 2016-06-07

**Authors:** Bing Li, Xinyi Zhu, Jianhua Hou, Tingji Chen, Pengyun Wang, Juan Li

**Affiliations:** ^1^Center on Aging Psychology, Key Laboratory of Mental Health, Institute of Psychology, Chinese Academy of SciencesBeijing, China; ^2^English Department, Faculty of Humanities and Educational Sciences, Technische Universität BraunschweigBraunschweig, Germany; ^3^University of Chinese Academy of SciencesBeijing, China; ^4^Human Information Processing Laboratory, School of Social Science and Humanities, University of TampereTampere, Finland; ^5^State Key Laboratory of Brain and Cognitive Science, Institute of Biophysics, Chinese Academy of SciencesBeijing, China

**Keywords:** cognitive training, memory strategy training, executive function training, older adults, mnemonic utilization

## Abstract

As mnemonic utilization deficit in older adults associates with age-related decline in executive function, we hypothesized that memory strategy training combined with executive function training might induce larger training effect in memory and broader training effects in non-memory outcomes than pure memory training. The present study compared the effects of combined cognitive training (executive function training plus memory strategy training) to pure memory strategy training. Forty healthy older adults were randomly assigned to a combined cognitive training group or a memory strategy training group. A control group receiving no training was also included. Combined cognitive training group received 16 sessions of training (eight sessions of executive function training followed by eight sessions of memory strategy training). Memory training group received 16 sessions of memory strategy training. The results partly supported our hypothesis in that indeed improved performance on executive function was only found in combined training group, whereas memory performance increased less in combined training compared to memory strategy group. Results suggest that combined cognitive training may be less efficient than pure memory training in memory outcomes, though the influences from insufficient training time and less closeness between trained executive function and working memory could not be excluded; however it has broader training effects in non-memory outcomes. Clinical Trial Registration: www.chictr.org.cn, identifier ChiCTR-OON-16007793.

## Introduction

Aging is associated with brain changes and cognitive decline that could limit older adults' functional capability (Lustig et al., 2009; Bishop et al., 2010; Mitchell et al., 2010; Talmelli et al., 2010). Encouragingly, accumulating evidence has shown that older adults reserve cognitive and brain plasticity, the capacity of relatively long-lasting neurological changes in response to experience (Lampit et al., 2015). Different formats of cognitive intervention, from standardized cognitive training (e.g., Jackson et al., 2012) to cognitive stimulation (e.g., Tesky et al., 2011), have shown beneficial effects in maintaining or even promoting cognitive functioning for both healthy older adults (for reviews see Kelly et al., 2014; Lampit et al., 2015) and elderly with cognitive impairments (for a review see Stott and Spector, 2011).

Memory deficits is one of the major manifestations of cognitive aging, representing both objective declines (Nyberg et al., 1996; Rabbitt and Lowe, 2000; Salthouse, 2004) and subjective concerns (Jonker et al., 2000; Perrig-Chiello et al., 2000). Memory training, which targets on improving episodic memory performance, has been frequently administrated in the elderly population (e.g., Derwinger et al., 2003; Cavallini and Bottiroli, 2009). Memory training enhanced memory performance by teaching memory strategies which facilitates information encoding and retrieval. Mnemonic strategies mainly include method of loci, face-name mnemonics, categorization, association, visual imagery, rehearsal, and so forth (for a review see Gross et al., 2012). The overall training effects of memory training on both objective and subjective measures of episodic memory have been convincingly proved by various studies, including large sample trials as the Advanced Cognitive Training for Independent and Vital Elderly (Ball et al., 2002). Ball et al. (2002) trained a large number of older adults (*n* = 711) with multiple mnemonics and found that the participants still benefited from the training in a 10-year follow-up. A recent meta-analytic study shows that memory strategy training for older adults induced a moderate training effect on overall episodic memory function (Cohen's *d* = 0.31) compared to control groups (Gross et al., 2012).

Nevertheless, memory strategy training has its limitation in two aspects. First, mnemonic utilization is difficult for older adults even after memory training. Several studies have found that age differences in memory performance were magnified rather than reduced after mnemonic training (Kliegl et al., 1989; Baltes and Kliegl, 1992; Nyberg et al., 2003). It has been argued that age-related executive function decline might hinder older adults from utilizing mnemonic techniques efficiently (Jones et al., 2006). For example, Nyberg et al. (2003) investigated brain activation differences between younger and older adults using the method of loci strategy. The results showed that when contrasting post- and pre-test, younger adults improved more in the trained memory task than older adults. Moreover, the younger participants showed increased activity in the left dorsolateral prefrontal cortex (DLPFC) during loci utilization compared to baseline conditions, which was not observed in older participants. Kirchhoff et al. (2014) investigated the role of prefrontal cortex in observed age differences in self-initiated strategy use. The results demonstrated that age differences in prefrontal gray matter volume significantly influence the use of self-initiated elaborative memory strategy use. Prefrontal cortex activation was reported in different forms of mnemonic processing such as association, binding, and mental image (Petrides, 2002; Bunge et al., 2004; Jha et al., 2006; Zeithamova and Preston, 2010). Taken together, these results implied that the mnemonic utilization deficit in the elderly may be due to age-related executive function decline. It is possible that conducting executive function training before memory strategy training would promote mnemonic application and lead to larger training gains in memory for older adults. The second limitation of memory strategy training is limited transfer effects (Singer et al., 2003; Carretti et al., 2007). Transfer effects in other cognitive domains were rarely reported in the mnemonic training literature with healthy older adults. Only a few studies reported improvement in other cognitive functions beyond episodic memory, such as in processing speed (Singer et al., 2003) or working memory (Carretti et al., 2007). Compared to memory strategy training, processed-based cognitive training, such as executive function training and working memory training, has relatively larger transfer effects (Zelinski, 2009). Recent meta-analyses and systematic reviews have shown that executive function induced moderate to medium training effects on overall cognition and moderate transfer effects (Karbach and Verhaeghen, 2014). Transfer effects have been reported in training studies on different types of executive functions (for a review see Strobach et al., 2014). In specific, task switching training may lead to improvements in untrained Stroop task and working memory tasks (Karbach and Kray, 2009; Kray et al., 2012) and updating tasks (Zinke et al., 2014). Updating training may induce near transfer to structurally similar working memory updating tasks (Dahlin et al., 2008; Li et al., 2008) and far transfer to task switching (Salminen et al., 2012) and fluid intelligence (Jaeggi et al., 2008).

Multi-component cognitive trainings include complex cognitive process yielding broader effect on multiple cognitive domains than single domain training (Cheng et al., 2012; Walton et al., 2015). However, to our knowledge no study has experimentally explored the comparative effect between executive function plus memory training and pure memory strategy training. We aimed at combining memory strategy training with executive function training to compose a combined training regimen, which was supposed to facilitate the efficiency of memory strategy training. Because mnemonic utilization deficit in older adults related to executive function decline, executive function training was arranged before mnemonic training in this combined training regimen. We assume that the preceding executive function training would improve executive function, and the hypothetically improved executive function would facilitate the effective utilization of memory strategy in older adults, thus resulting in larger gains from subsequent memory strategy training. In this study, we compared combined training to pure memory strategy training. Although the duration of memory strategy training component in combined training group was only half of that in pure memory training group, we expected similar or even larger improvements in memory in the combined group because we assumed better strategy utilization after executive function training in the combined group. In addition, we also predicted broader effects in the combined group, for executive function training would improve performance on both trained and untrained executive function tasks. Specifically, as executive function training included updating and task switching training, we predicted combined group would show improvements in trained updating and switching tasks, as well as in untrained tasks including the Stroop Test and the Trail Making Test. To summarize, we hypothesized that in a fixed training interval this combined cognitive training regimen would be more efficient than pure memory strategy training. That is, it would induce (1) comparable or even larger improvements in memory, and (2) increased performance in executive function and other cognitive domains.

## Methods

### Participants

**Figure 1:**
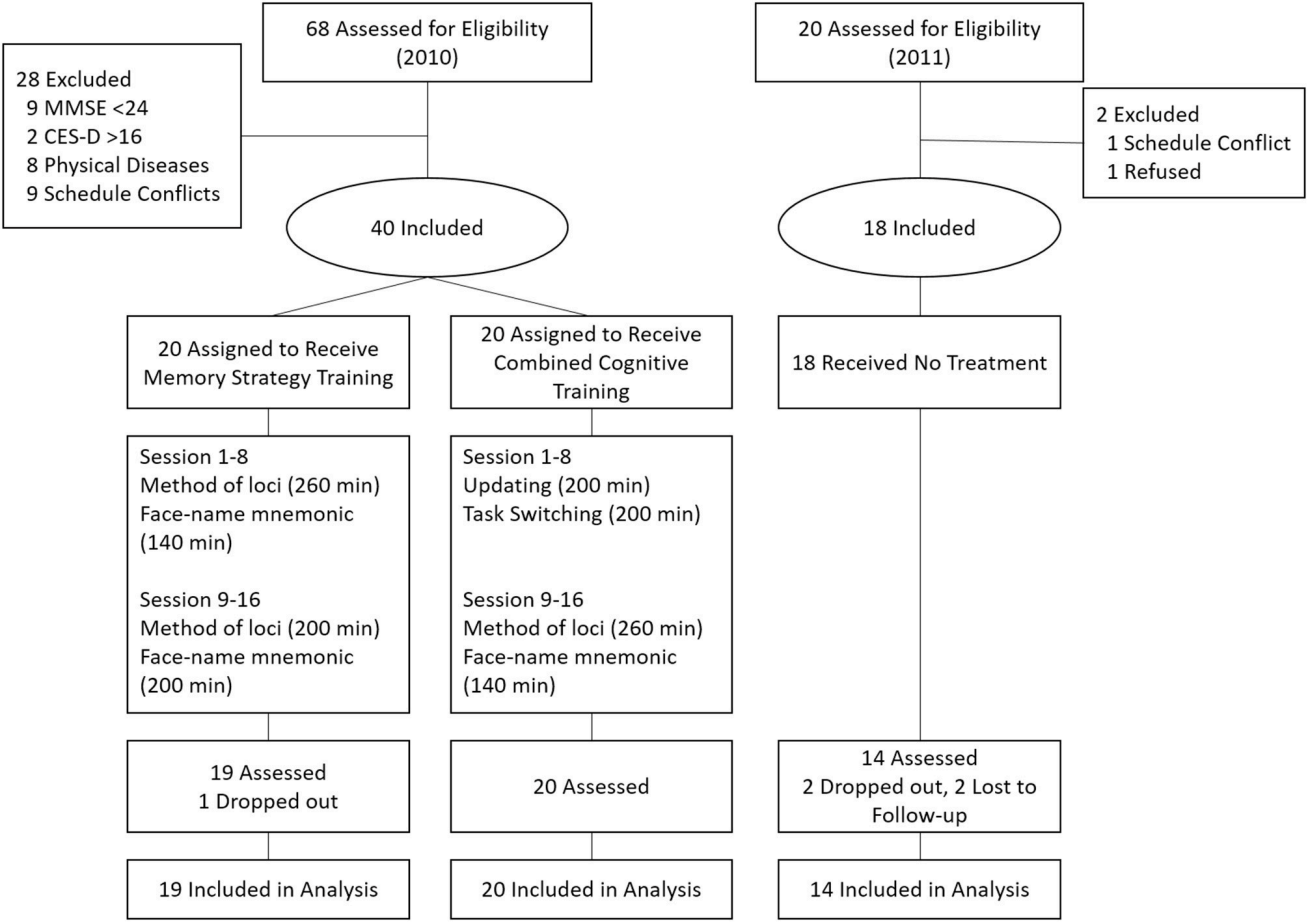
**Flow of participants**.

The participants were recruited by advertisements on the community bulletin and flyers in the community service station. A group of 68 older adults from the local community were voluntarily contacted in April, 2010. We excluded 28 older adults who (1) scored less than 24 on Mini-Mental State Examination (MMSE) (*n* = 9); (2) scored more than 16 on Center for Epidemiological Studies Depression Scale (CES-D) score (*n* = 2); (3) had visual or hearing impairments which would hinder from training (*n* = 8); (4) could not guarantee a full participation because of scheduling conflicts (*n* = 9). Thus, 40 participants (mean age = 68.3, *SD* = 4.4, range 60–76) were randomly assigned to a memory strategy training group (memory group) or a combined cognitive training group (combined group). One participant in memory group who dropped out before post-test was excluded from analysis. Another group of 18 participants were recruited as a passive control group under the same inclusion criterion in October, 2011. They received no training but only the pre- and post-test. At post-test, four participants dropped out (two participants withdrew from the study, and two could not be contacted). Thus, fourteen participants in the control group were included in analysis. At follow-up, two participants in the memory group and six in combined group dropped out. As seen in Table 1, the three groups of participants did not differ significantly (*p* > 0.05) in age, gender, years of education, global cognition (MMSE), and depression (CES-D). Participants gave informed consent and were paid 200 Yuan (about 30 dollars) for participation. Figure 1 shows the flow of participants.

**Table 1:** **Demographic and cognitive characteristics**.

	**Memory (*n* = 19) M (*SD*)**	**Combined (*n* = 20) M (*SD*)**	**Control M (*SD*)**	***P*-value (*n* = 14)**
Age (years)	67.8 (4.3)	68.7 (4.6)	67.6 (4.2)	0.76
Education (years)	13.2 (2.8)	12.8 (3.3)	13.3 (2.7)	0.84
Female/Male	15/4	11/9	10/4	0.26
MMSE	28.3 (1.1)	28.0 (1.5)	28.5 (1.3)	0.49
CES-D	6.1 (5.4)	7.6 (9.0)	5.0 (5.8)	0.58

*MMSE, Mini Mental State Examination; CES-D, Center for Epidemiological Studies Depression Scale*.

### Procedure

All three groups of participants received cognitive assessments before and after training. Memory group and combined group were also assessed at 4 month after completion of training. Follow-up data of control group was not collected. Cognitive assessments at each time were administrated in one session by assessors who were blind to training assignment. The assessment session lasted about 90–120 min. The pre-training assessments were conducted within 2 weeks before training, and the post-training assessments were completed within a week after the final session of training.

After baseline test, participants in the memory group and combined group received 16 sessions of training during about 6 weeks. Training was held three times per week. Each session lasted for ~60 min (50 min for training and 10 min for break). Thus, each training group received 16 h of training in total. Frequency, time and format of training were matched in two groups. In memory group, participants received 16 sessions of memory strategy training, including training for method of loci (460 min in total) and face-name mnemonic (340 min in total). In combined group, participants firstly received eight sessions of executive function training (200 min for updating and 200 min for switching) and then eight sessions of memory strategy training (260 min for method of loci and 140 min for face-name mnemonic). Executive function training was arranged before memory strategy training because we assumed that enhanced executive function induced by preceding executive function training would facilitate the effective utilization of memory strategy, making the subsequent memory strategy training more efficient. The protocol of this study was approved by the Ethics Committee of the Institute of Psychology, Chinese Academy of Sciences. The study was registered in the Chinese Clinical Trial Registry (www.chictr.org.cn) ChiCTR-OON-16007793.

### Memory strategy training

#### Method of loci

Participants practiced method of loci (Bower, 1970) in the Wordlist task. They were instructed to establish a well-known route with several landmarks and to associate words with those landmarks serially on a mental map. At recall, participants were instructed to mentally revisit the ordered landmarks to retrieve the words. In this study, the Wordlist task words were names of common objects (e.g., animals, fruits) and were read aloud to participants. The method of loci practice started from eight-word lists, and then gradually increased to 10-, 12-, 14-, and 16-word lists. During the final 9–16 sessions, participants practiced only 16-word lists.

#### Face-name mnemonic

The face-name mnemonic (Yesavage, 1983) was used in the Face-name task. In this task, black-and-white photos of males or females with two-character Chinese names were visually presented to participants on a computer. They were instructed to identify a prominent facial feature, creating a visual association with the name, and then mentally connect the association with the prominent facial feature. At recall, participants needed to identify the prominent feature first, retrieve the related mental image, and then recall the name. For the first eight memory strategy training sessions, participants progressively practiced the face-name mnemonic on one-, two-face, and three-face tasks. During sessions 9–16, participants kept practicing on five- and seven-face tasks.

### Executive function training

#### Updating training

The trained updating task was the keep-track task (Yntema and Mueser, 1962). In this study, the keep-track task was divided into Word-updating and Picture-updating tasks. Both were involved equally in training sessions. In this task, trials of colored words and pictures from various semantic categories (e.g., animals, clothes, vegetables, and fruits) were presented serially at 2000 ms/per item with an interval of 500 ms in random order. Participants were instructed to continuously update the items of targeted categories indicated by boxes at the bottom of the screen and verbally report the last item of each targeted category at the end of the trial. Each trial included two distracting items beyond the targeted categories. Each task included 12 trials of words or pictures. Difficulty was manipulated by varying the number of categories presented (two or three) and the number of items in each category (two, three, or four). Participants started with the easiest task (two categories with two items per category). When easier tasks were finished, they were instructed to conduct the more difficult tasks along with training sessions.

#### Switching training

We used the task-switching paradigm (Kray and Lindenberger, 2000) modified to include mixed-task blocks only (two- and three-task switching). In this task, participants were required to switch subtasks (two subtasks or three subtasks) on pseudorandom trials according to cues presented in two boxes at the bottom of the screen in each trial. Two-task switching training consisted of two types of training materials performed equally in the training phase—“food” and “poker.” For the “food” task, subtask A required participants to decide whether a picture showed a fruit or vegetable (fruit/vegetable), and subtask B whether a picture was on the left or the right of the screen (left/right). For the “poker” task, participants were required to decide whether a poker card depicted was red or black (red/black), or whether the number on the poker card was even or odd (even/odd). There were also two sorts of training materials performed equally in three-task switching training —“face” (male/female, elders/youngsters, and white/yellow) and “poker” (red/black, even/odd, and < 5/>5). The same two response keys were used for all tasks. Difficulty was manipulated by varying the number of subtasks (two and three) and the number of items in each subtask (8, 10, and 12). The number of trials in each task varied according task difficulty, ranging from 16 trials (the easiest task) to 36 trials (the hardest task). Participants started with the easiest task (two subtasks with eight items per subtask) and continued to more difficult ones along with training sessions.

### Outcome measures

#### Trained memory tasks

For the Wordlist task and the Face-name task, two versions were used at pre- and post-test. These versions were assessed by a group of 15 healthy elderly adults who did not take part in our training, and the results of free verbal recall within each version did not differ significantly from each other. In follow-up, we used the pre-test version.

#### Wordlist task

An audio-taped list of 16 two-character words was presented to participants at a rate of 6 s per word. At the end of the presentation participants were required to verbally recall as many words on the list as possible with no order constraint. The words were from four semantic categories (fruits, vegetables, animals, clothes) with four words per category and were chosen from the Directed Memory Test (from the Clinical Memory Scale; Xu and Wu, 1986). The dependent measure was the number of correctly recalled words (maximum score = 16).

#### Face-name task

Participants were presented with 12 black-and-white photographs of faces balanced for sex and paired with two-character Chinese names at the bottom of the screen. Each photograph was shown on the computer for 30 s. At recall, these faces were presented in a different order, and participants were asked to verbally report the names previously paired with those faces. The dependent measure was the number of correctly recalled names (maximum score = 12).

### Non-trained memory tasks

#### Associative learning test (ALT, from the clinical memory scale, Xu and Wu 1986)

Participants were required to study an audio-taped list of 12 pairs of nouns for 2 s per pair. Half of the word pairs were not associated (e.g., teacher-railway) and half were semantically associated (e.g., sun-moon). The list was presented three times with different orders. After each study phase, participants were asked to recall the second noun when given the first noun as a cue after each trial. The cues were not presented in the same order as during the training phase. The number of correctly recalled nouns was the dependent measure (0.5 point per pair for semantically-associated pairs, one point per pair for non-associated pairs, maximum = 27).

#### Logical memory test (LMT, from the wechsler memory scale-revised in China; Gong, 1989)

Participants were presented with two audio-taped stories and were asked to verbally recall a story immediately after its presentation. The dependent measure was the mean score of the number of correctly recalled episodes (50 in total) of two stories (maximum score = 25).

#### Multifactorial memory questionnaire (MMQ, Troyer and Rich, 2002)

This questionnaire was used to assess separate dimensions of subjective memory, including contentment, ability and strategy. It contains 57 items addressing a variety of subjective perception of participants' current memory ability. In this study, only the MMQ-Ability Scale (20 items) was used to assess the memory failures in everyday memory situations, such as remembering names and appointments. Participants reported the frequency of each memory failure on a five-point scale (never, rarely, sometimes, often, all the time). Answers on each item were recoded to zero to four points based on the reported frequency. The sum score indexed subjective memory, with higher scores indicating more memory failures.

### Trained executive function tasks

#### Word/picture updating task

Word- and Picture-updating tasks were structurally similar to the trained updating tasks. There were six two-category trials (half three items/category, half four items/category) and six three-category trials (half two items/category, half three items/category) in this task with two distracting items in each trial. The dependent measure was the number of blocks where the last presented items of each category were correctly recalled (maximum score = 12).

#### Switching task

Switching task was structurally similar to the trained “poker” switching task. This task was three-task switching with 24 items per subtask. Switching costs, the reaction time difference between non-switch and switch items, were measured as dependent variables. Each trial began with fixation-cross presentation (2000 ms) and did not advance until participant response. Participants were instructed to respond as quickly and accurately as possible.

### Non-trained executive function tasks

#### Trail making test (B–A)

The Trail Making Test (TMT, Reitan, 1955) included two parts. Part A is a neuropsychological test of processing speed and part B is a test of switching. Performance was indexed by the difference value between the reaction time on TMT-B and time on TMT-A (TMT B–A). Larger values in TMT (B–A) indicated poorer performance.

#### Stroop test (Stroop, 1935)

Participants were asked to name the color of dots/words/color words in three cards. Performance was indexed by the time difference between the card of color words and the card of dots. Larger values indicated poorer performance.

#### Backward digit span task (from the wechsler adults intelligence scale-revised in China; Gong, 1992)

Participants were asked to reversely repeat lists of digits after each auditory presentation. The digit span was the length of the longest list a participant could repeat.

### Processing speed, short-term memory, and language tasks

Performance on TMT-A was used to measure processing speed. The Forward Digit Span Task (from the Wechsler Adults Intelligence Scale-Revised in China) was adopted to measure short-term memory. The Verbal Fluency Test (González et al., 2005) was used to measure language abilities. Participants were asked to verbally report as many animals or foods as possible to assess category fluency with this test.

### Analysis

First, one-way analyses of variance (ANOVA) were conducted to compare the baseline performance on cognitive tasks in three groups. Significant differences (*p* < 0.05) were found on the face-name task, ALT, word updating task and backward digit span, and marginally significant difference (*p* < 0.1) was found on the picture updating task. Thus, to control the discrepant baseline performance, analyses of covariance (ANCOVA) of post-training-minus-baseline change scores were used to compare training effects among three groups, using baseline scores on these five tasks as covariates and group as a factor. ANCOVA was performed for each outcome measure, including four tasks of objective memory, MMQ, and five tasks of executive function. A composite score of objective memory measures was calculated to index the overall memory performance. Raw scores on four trained and untrained memory tasks (the wordlist task, face-name task, ALT, and LMT) were standardized into *z* scores, and then summed and converted (equally weighted) into a composite *t*-score (mean = 50, standard deviation = 10). Similarly, a composite score of executive function was computed, combining performance on word, and picture updating tasks, the Stroop Test, TMT and backward digit span. In addition, effect sizes (Cohen's *d*; Cohen, 1988) for repeated measures (baseline vs. post-training) of all outcome measures and composite scores within each group were also calculated. Baseline data of switching task from eight participants in the control group was lost due to technique problem. Because of high missing rate of data, switching task was not included in the analysis.

Although follow-up data was not available for control group, maintenance effects were analyzed based on data from two training groups. As no significant difference was found between two training groups at baseline, 2 (group: combined group vs. memory group) × 2 (time: baseline vs. follow-up) ANOVAs were used to compare changes from baseline to 4-month follow-up in two training groups.

## Results

### Training effects on memory

**Figure 2:**
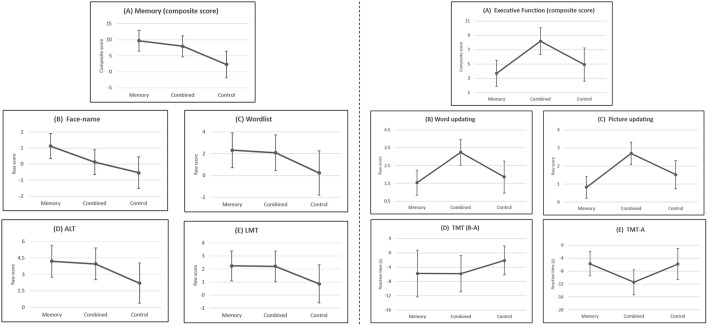
**Estimated means of change scores with 95% confidence interval on memory (left) and executive function tasks (right)**. Memory tasks: **(A)** Composite score of memory; **(B)** Face-name task; **(C)** Wordlist task; **(D)** Associative Learning Test; **(E)** Logical Memory Test. Higher scores indicates greater improvements. Executive function tasks: **(A)** Composite score of executive function; **(B)** Word updating task; **(C)** Picture updating task; **(D)** Trail Making Test (B–A); **(E)** Trail Making Test-A. In **(A)**, **(B)**, and **(C)**, higher scores indicate greater improvements. In **(D)** and **(E)**, lower scores indicate greater improvements.

Baseline cognitive performance, estimated means of change scores, effect sizes (Cohen's *d*) and significance were shown in Table 2. The ANCOVA results showed significant effects of group on the measure of face-name task and composite score of memory (Figure 2). Further analysis showed that, compared to control group, memory group demonstrated significant improvement on the face-name task (*p* < 0.05) while combined group did not (*p* > 0.05). For composite score of memory, significant training effects was found in memory group (memory vs. control, *p* < 0.01), and marginally significant effect was found in combined group (combined vs. control, *p* = 0.05). The effect size analyses revealed memory group had larger effect sizes on all memory measures than combined group (see Table 2).

**Table 2:** **Post-training effects of training in three groups**.

	**Memory (*n* = 19)**			**Combined (*n* = 20)**			**Control (*n* = 14)**			***P*^b^**	**Partial eta squared**
	**Baseline mean (*SD*)**	**Change mean (95% CI)**	**Effect size**	**Baseline mean (*SD*)**	**Change mean (95% CI)**	**Effect size**	**Baseline mean (*SD*)**	**Change mean (95% CI)**	**Effect size**		
**MEMORY**											
Memory (composite score)	50.71 (7.46)	9.66 (6.40–12.91)	1.189	47.35 (6.84)	7.92 (4.62–11.22)	0.833	52.82 (5.32)	2.26 (−1.89–6.41)	0.486	0.030	0.15
**TRAINED MEMORY TASKS**											
Face-name	3.5 (1.75)	1.12 (0.35–1.90)	0.504	2.70 (2.04)	0.12 (−0.67–0.90)	0.016	4.54 (2.10)	−0.54 (−1.53–0.44)	−0.16	0.030	0.14
Wordlist	9.89 (2.49)	2.32 (0.72–3.92)	0.972	9.00 (2.22)	2.09 (0.47–3.71)	0.811	9.21 (1.89)	0.23 (−1.81–2.26)	0.219	0.271	0.06
**NON-TRAINED MEMORY TASKS**											
ALT	10.29 (3.73)	4.20 (2.76–5.64)	1.179	10.08 (2.17)	3.95 (2.50–5.40)	1.164	12.5 (2.69)	2.20 (0.37–4.03)	0.764	0.233	0.06
LMT	8.79 (2.75)	2.24 (1.09–3.40)	0.864	7.60 (2.39)	2.20 (1.02–3.37)	0.672	8.61 (2.04)	0.86 (−0.62–2.33)	0.44	0.316	0.05
MMQ	16.89 (10.73)	−2.75 (−6.40–0.90)	0.275	20.25 (10.01)	0.70 (−3.07–4.46)	−0.049	16.38 (7.65)	0.57 (−4.33–5.47)	−0.073	0.349	0.05
**EXECUTIVE FUNCTION**											
EF (composite score)	51.33 (5.82)	3.69 (1.86–5.53)	0.653	46.12 (7.83)	8.19 (6.33–10.04)	1.22	53.74 (7.62)	4.91 (2.57–7.25)	0.52	0.005	0.21
**TRAINED EF TASKS**											
Word updating	5.37 (1.71)	1.53 (0.83–2.24)	0.971	3.80 (2.35)	3.24 (2.52–3.95)	1.659	6.07 (2.53)	1.86 (0.96–2.76)	0.523	0.005	0.21
Picture updating	7.58 (1.74)	0.82 (0.21–1.44)	0.338	6.05 (2.24)	2.70 (2.08–3.33)	1.449	7.21 (2.12)	1.52 (0.74–2.30)	0.787	<0.001	0.29
**NON-TRAINED EF TAKS**											
TMT (B–A)^a^	25.89 (17.08)	−5.76 (−12.28–0.77)	0.74	29.80 (18.65)	−5.84 (−12.44–0.77)	0.869	23.50 (13.70)	−2.14 (−10.45–6.18)	0.352	0.766	0.01
Stroop test^a^	12.89 (6.23)	−1.30 (−6.14–3.53)	0.143	10.20 (12.69)	2.37 (−2.52–7.26)	0.053	17.5 (8.42)	−5.97 (−12.13–0.19)	0.529	0.150	0.08
Digital span backward	4.9 (1.24)	0.13 (−0.34–0.60)	0.172	4.25 (1.16)	0.37 (−0.10–0.85)	0.376	5.86 (1.41)	0.43 (−0.16–1.03)	0.113	0.661	0.02
**OTHERS COGNITIVE TASKS**											
Digital span forward	7.89 (1.15)	−0.20 (−0.48–0.88)	0.228	7.50 (1.15)	−0.09 (−0.79–0.60)	−0.077	7.93 (1.33)	0.86 (−0.01–1.73)	0.658	0.275	0.06
TMT-A^a^	32.53 (6.11)	−5.74 (−9.53–1.95)	1.083	36.70 (10.27)	−11.45 (−15.29–7.62)	1.304	34.21 (10.67)	−5.85 (−10.68–1.01)	0.477	0.087	0.10
Verbal fluency	48.05 (8.35)	6.51 (1.89–11.12)	0.834	45.05 (9.24)	5.78 (1.11–10.46)	0.643	50.36 (12.28)	3.91 (−1.98–9.79)	0.225	0.793	0.01

*SD, standard deviation; CI, confidence interval; ALT, Associative Learning Test; LMT, Logical Memory Test; EF, executive function; TMT, Trail Making Test; MMQ, Multifactorial Memory Questionnaire*.

a *Lower scores indicate better performance*.

b *Analyses of covariance of posttraining-minus-baseline change scores, controlled for baseline performance on the face-name task, ALT, word updating task and backward digit span, and picture updating task*.

### Training effects on executive function and other cognitive abilities

For executive function, the ANCOVA results indicated significant effects of group on the measure of word and picture updating tasks (Figure 2). Significant improvements were found in combined group (combined vs. control, *p* < 0.01, for word updating task; *p* < 0.001, for picture updating task) but not memory group (memory vs. control, *p* > 0.05, for word updating task; *p* > 0.05, for picture updating task). Improvements on the composite score of executive function also showed significant group differences. Further analysis indicated significant improvement in combined group (combined vs. control, *p* < 0.05) but not memory group (memory vs. control, *p* > 0.05).

The ANCOVA results also revealed a marginal significant effect of group on TMT-A (Figure 2). The combined group showed a trend of improvement on TMT-A (combined vs. control, *p* = 0.09) while memory group did not.

### Maintenance effects

Results of maintenance effects based on data from two training groups were shown in Table 3. The ANOVAs showed no significant group × time interactions on all outcome measures. The main effects of time were significant on measures of face-name task, wordlist task, ALT, LMT, word and picture updating tasks, TMT-A, and verbal fluency, indicating improved performance at 4-month follow-up in both training groups.

**Table 3:** **Cognitive performance of two training groups at the 4-month follow-up**.

	**Memory (*n* = 17)**				**Combined (*n* = 14)**				***P*-value^b^**	**Partial eta squared**
	**Basline**		**Follow-up**		**Basline**		**Follow-up**			
	**Mean**	***SD***	**Mean**	***SD***	**Mean**	***SD***	**Mean**	***SD***		
**TRAINED MEMORY TASKS**										
Face-name	3.65	1.79	6.76	2.62	2.75	1.92	4.89	2.63	0.20	0.06
Wordlist	10.06	2.59	12.59	2.79	9.14	1.56	10.43	2.87	0.17	0.06
**NON-TRAINED MEMORY TASKS**										
ALT	10.59	3.79	15.62	4.65	10.11	2.47	14.39	3.13	0.59	0.01
LMT	9.00	2.55	10.38	2.97	8.14	2.20	9.14	2.67	0.71	0.01
MMQ	15.24	9.66	15.00	8.14	20.79	10.89	24.00	11.5	0.22	0.05
**TRAINED EF TASKS**										
Word updating	5.47	1.70	7.47	1.94	3.57	2.21	7.00	2.83	0.11	0.09
Picture updating	7.65	1.80	8.47	1.66	6.07	1.90	8.43	2.31	0.07	0.11
**NON-TRAINED EF TAKS**										
TMT (B-A)^a^	27.00	17.51	21.12	15.50	29.07	20.44	27.36	18.30	0.52	0.01
Stroop test^a^	12.00	5.51	13.76	7.66	12.14	13.83	10.64	7.74	0.34	0.03
Digital span backward	5.00	1.28	5.53	1.74	4.14	1.10	4.43	1.83	0.62	0.01
**OTHERS COGNITIVE TASKS**										
Digital span forward	7.76	1.09	7.71	1.05	7.50	1.09	7.43	1.34	0.98	0.00
TMT-A^a^	32.47	6.48	26.29	8.56	36.43	8.43	27.36	6.27	0.37	0.03
Verbal fluency	49.00	8.30	53.12	11.29	44.93	8.68	48.93	9.26	0.97	0.00

*SD, standard deviation; CI, confidence interval; ALT: Associative Learning Test; LMT: Logical Memory Test; EF, executive function; TMT: Trail Making Test; MMQ, Multifactorial Memory Questionnaire*.

a *Lower scores indicate better performance*.

b *P-values for 2 (combined group vs. memory group) × 2 (baseline vs. follow-up) ANOVAs*.

## Discussion

The present study compared the effectiveness between combined cognitive training (executive training plus memory training) and memory strategy training. In respect of composite score of memory outcomes, memory group exerted significant improvements than control group while combined group exerted marginally significant enhancement. In respect of non-memory outcomes, improvements on executive function and processing speed were only found in the combined training group but not memory training group.

We hypothesized that combined cognitive training group would exert comparable or even larger improvements in memory than memory training group. Compared with control group, significant training effect was detected in memory tasks and marginally significant training effect was found in combined cognitive training group. Contrary to our expectation, combined cognitive training did not even provide comparable training gains in memory function than memory strategy training alone; rather, the gains were smaller. As our results demonstrate, this objective is not achieved. One possible explanation for this is that executive function training time may be insufficient to effectively improve mnemonic use. Executive function training is based on extensive practice, and its mechanisms leading to training effects are associated with continuous performance of the trained abilities, so training time affects the practice-based processing changes (Zelinski, 2009). According to previous studies, Dahlin et al. (2008) trained participants for 11.25 h on updating tasks and found episodic memory enhancement exclusively in younger adults; Buschkuehl et al. (2008) trained participants for 17.25 h on similar updating tasks and observed an increase in episodic memory in old-old adults. In our study, there were only 4 h of updating task training and 4 h of switching task training. In addition, we trained subjects on executive function instead of directly on working memory, while previous neuroimaging studies showed that working memory contributed to memory organizational skills (D'Esposito et al., 1999; Petrides, 2002; Blumenfeld and Ranganath, 2006), and we did not detect transfer effect on working memory. Further, because memory training time in combined cognitive training group was only half of that in memory group, intervention gain could possibly be more difficult to detect.

Consistent with our expectation, combined cognitive training group demonstrated broader effect in executive function compared with memory training group alone. The improvements in executive function as revealed by Word- and Picture-updating task were in line with previous findings that have reported considerable gains in trained updating or switching tasks (Dahlin et al., 2008; Minear and Shah, 2008; Karbach and Kray, 2009; Borella et al., 2010). For the processing speed improvement, transfer effect was found in Trail Making Test-A which is typical processing speed test in combined cognitive training group. The transfer to processing speed was reported in Singer's et al. (2003) study, which used a processing speed task, the Digit Symbol Test, different from the Trail Making Test-A used in this study. In short, analyses in this study provided more evidence for the exciting idea that combined cognitive training may be capable of improving multi-domain cognitive functions.

Several limitations of the present study should be noted. First, baseline performances were not matched on face-name task, ALT, word and picture updating tasks, and backward digit span. This may due to non-randomization of three groups. Two training groups were randomized, while data of control group was collected a year after the completion of two training regimens. Although baseline performance on these five tasks was treated as covariate in the analyses, we cannot exclude the confounding effects of unmatched baseline performance. Second, the sample size of our study was rather small, leading to limit statistical power for testing hypothesis (Shieh, 2014). Third, follow-up data of the control group was not collected, so whether training gains maintained over time could not be clearly investigated. The results revealed that both training groups showed improved performance on trained and untrained memory tasks, trained EF tasks, and tests of processing speed and language at follow-up compared to baseline. However, the results must be cautiously interpreted because both maintenance effect and practice effect could contribute to performance at follow-up. Nonetheless, this study can contribute to the gerontological literature in that it experimentally analyzed how different components of combined cognitive training interact with each other.

In sum, our main outcomes contribute to the intervention literature in three ways. First, combined cognitive training provided smaller improvement in memory, but broader effects on other cognitive functions. Therefore, it would be a wise choice for combined cognitive intervention in clinical therapy for patients with multi-domain deficit. Second, memory strategy training uniquely improved memory with regard to immediate training effects. For future research, especially for research in clinical therapy for memory deficient patients (e.g., MCI), it is necessary to further investigate whether memory strategy training would provide better training effects to memory-impaired older adults compared to other combined cognitive training regimens. Third, we experimentally explored how executive function training may influence memory training. Given several limitations in this study, further investigations are needed to test how to effectively arrange different components in interventional studies to meet different purposes.

## Author contributions

BL and JL initiated and designed the study. BL and PW developed the detailed protocol. BL administrated the training program. XZ, TC, and PW contributed to data collection. BL and XZ analyzed the data. BL, XZ, JH, and JL wrote the paper. JL supervised the study.

### Conflict of interest statement

The authors declare that the research was conducted in the absence of any commercial or financial relationships that could be construed as a potential conflict of interest.
